# Validation of the Posture Analyzing and Virtual Reconstruction (PAViR) System for Measuring the Hip–Knee–Ankle Angle Using 2D Photogrammetry and Computer Vision

**DOI:** 10.3390/diagnostics16040568

**Published:** 2026-02-13

**Authors:** Carmen Aguilar Esteban, Elena Martinez Mendoza, Carla Martinez Navarro, Javier Torralba Estelles

**Affiliations:** 1 Private Practice at dePie Clínicas Podológicas, 46021 Valencia, Spain; 2 Department of Podiatry, School of Medicine and Health Science, Catholic University of Valencia, 46900 Torrent, Spain

**Keywords:** Hip–Knee–Ankle angle, PAViR, 2D photogrammetry, biomechanics, kinematic analysis, computer vision, lower limb alignment

## Abstract

**Background**. Accurate assessment of lower limb alignment is critical in diagnostic decision-making for musculoskeletal disorders. This study aimed to validate the PAViR (Posture Analyzing and Virtual Reconstruction) system, a non-invasive device based on artificial vision and 2D photogrammetry, for measuring the Hip–Knee–Ankle (HKA) angle. **Method**. A total of sixty-one adult participants were evaluated using the PAViR system, and the results were compared against Kinovea, a validated open-source software commonly used for 2D kinematic and angular analysis in clinical and sports biomechanics. Statistical analyses included the Shapiro–Wilk test, Pearson correlation, and Bland–Altman plots. **Results**. The correlation between both systems was perfect (r = 0.999; *p* < 0.001). The Bland–Altman analysis showed differences of 0.03° (left) and 0.04° (right), with limits of agreement between −0.25° and +0.75°, within the clinically acceptable margin of ±2°. These findings demonstrate that the PAViR system shows excellent agreement with a validated 2D photogrammetry reference method for measuring the Hip-Knee-Ankle angle in asymptomatic adults. The narrow limits of agreement (−0.25° to +0.75°) and minimal systematic bias (0.03–0.04°) support the technical validity of PAViR for static coronal plane alignment assessment under controlled conditions. **Conclusions**. Further validation studies in clinical populations and dynamic contexts are necessary to establish broader applicability and clinical utility. Its integration could enhance lower limb assessment in orthopedic, sports, and preventive care. Further validation studies in clinical populations with musculoskeletal pathology, dynamic functional contexts, and direct comparison with radiographic gold standards are necessary to establish broader applicability and clinical utility.

## 1. Introduction

Accurate assessment of lower limb alignment is fundamental in the diagnosis, monitoring, and treatment planning of musculoskeletal disorders, particularly those involving the knee joint. Among the available biomechanical parameters, the Hip–Knee–Ankle (HKA) angle is widely recognized as the most reliable indicator of coronal plane alignment of the lower limb and plays a central role in clinical decision-making across orthopedics, biomechanics, rehabilitation, and preventive medicine [1,2]. Deviations in HKA alignment have been strongly associated with altered joint loading patterns, disease progression in knee osteoarthritis, and outcomes following surgical interventions such as high tibial osteotomy or total knee arthroplasty [3,4].

Traditionally, the gold standard for HKA angle measurement has been full-length, weight-bearing radiography of the lower limbs. Although this technique provides high accuracy, its routine use is limited by several well-documented drawbacks, including exposure to ionizing radiation, relatively high costs, restricted accessibility, and logistical constraints in outpatient or non-hospital settings [5,6]. These limitations are particularly relevant in contexts requiring repeated measurements, such as longitudinal follow-up, screening programs, sports medicine, or preventive musculoskeletal care, where cumulative radiation exposure and practicality become critical concerns.

As a result, there has been growing interest in the development and validation of non-invasive, radiation-free alternatives capable of providing clinically acceptable accuracy for lower limb alignment assessment [7,8]. In recent years, advances in computer vision, artificial intelligence (AI), and photogrammetry have enabled the emergence of image-based systems that can extract biomechanical parameters from 2D or 3D visual data. These technologies have demonstrated promising results in posture analysis, gait assessment, and joint kinematics, offering scalable and accessible solutions for musculoskeletal evaluation [9,10,11].

Among these emerging tools, 2D photogrammetry combined with AI-assisted landmark detection has gained particular attention due to its balance between simplicity, cost-effectiveness, and clinical applicability. Previous studies have shown that, under standardized conditions, 2D image-based measurements can achieve levels of reliability and validity comparable to more complex motion capture systems for static alignment assessment [12,13,14]. However, despite these advances, robust validation of such systems for coronal plane alignment parameters—specifically the HKA angle—remains limited.

The Posture Analyzing and Virtual Reconstruction (PAViR) system represents a novel approach within this technological landscape. By integrating artificial vision algorithms with standardized image acquisition protocols, PAViR aims to identify anatomical landmarks automatically and compute alignment parameters in a fast, non-invasive manner. While prior investigations have demonstrated the validity of the PAViR system for sagittal postural assessment [15], its accuracy and agreement in measuring coronal plane alignment parameters such as the HKA angle have not yet been formally established.

Validating non-radiographic tools for HKA measurement is of particular clinical relevance. A reliable alternative to radiography could facilitate broader use of alignment assessment in primary care, rehabilitation, sports medicine, and preventive screening, as well as enable frequent monitoring without the risks associated with repeated radiation exposure. Furthermore, such tools may support early detection of malalignment-related risk factors and contribute to personalized musculoskeletal management strategies.

Therefore, the primary objective of this study was to validate the PAViR system for the measurement of the Hip-Knee-Ankle angle in asymptomatic adults by comparing its erformance with that of Kinovea, a validated 2D photogrammetry reference method. We hypothesized that the PAViR system would demonstrate a high level of agreement with the reference method, with differences remaining within clinically acceptable limits.

Establishing the validity of PAViR for HKA assessment represents a necessary preliminary step in evaluating the potential role of AI-assisted 2D photogrammetry for non-invasive lower limb alignment assessment in clinical settings. Future validation against the gold standard of full-length weight-bearing radiography will be necessary to establish absolute accuracy for detecting true anatomical alignment.

## 2. Materials and Methods

### 2.1. Study Design

This study followed a cross-sectional observational design aimed at validating a non-invasive imaging-based system for static lower limb alignment assessment. The primary outcome was the Hip–Knee–Ankle (HKA) angle measured in the coronal plane. Measurements obtained using the Posture Analyzing and Virtual Reconstruction (PAViR) The Posture Analyzing and Virtual Reconstruction (PAViR) system (commercially known as Moti Physio; Moti Physio Inc., Seoul, Republic of Korea) is a portable, modular device system were compared against those derived from Kinovea software (version 0.9.5; Kinovea Team, Bordeaux, France) which served as the reference method for 2D angular analysis.

The study was conducted in accordance with the Declaration of Helsinki and was approved by the Institutional Review Board of Universidad Católica de Valencia (protocol code UCV/2024-2025/028). All participants provided written informed consent prior to inclusion.

### 2.2. Participants

A total of 61 asymptomatic adult volunteers (41 males and 20 females) were recruited using non-probabilistic convenience sampling. Participants were eligible for inclusion if they met the following criteria: age between 20 and 45 years; absence of known musculoskeletal disorders affecting the lower limbs; no history of lower limb surgery; ability to maintain a stable bipedal standing position; and willingness to comply with the study protocol. All participants were of European Caucasian ethnicity, reflecting the demographic composition of the recruitment region. Participants were eligible for inclusion if they met the following criteria: age between 20 and 45 years; absence of known musculoskeletal disorders affecting the lower limbs; no history of lower limb surgery; ability to maintain a stable bipedal standing position; and willingness to comply with the study protocol.

Exclusion criteria included the presence of acute or chronic musculoskeletal pain, diagnosed lower limb deformities, prior orthopedic surgery, neurological or balance disorders, dermatological conditions that could interfere with landmark visualization, pregnancy, or any condition limiting the participant’s ability to maintain the standardized posture required for image acquisition.

The decision to include asymptomatic participants was based on the need to establish baseline validity and agreement under controlled conditions, minimizing confounding factors related to pain, compensatory postures, or severe anatomical alterations.

Participants were recruited between September 2024 and June 2025 using non-probabilistic convenience sampling from the university community and associated clinical settings in Valencia, Spain.

### 2.3. Sample Size Justification for Agreement Precision

Sample size was determined a priori to ensure adequate precision in the estimation of agreement between the PAViR system and the reference method, in accordance with Bland–Altman methodology. As Bland–Altman analysis does not involve hypothesis testing, concepts of statistical power or α/β error are not applicable. Instead, sample size was selected to achieve a sufficiently narrow 95% confidence interval (CI) around the es-timated limits of agreement (LoA). The half-width of the 95% CI for each limit of agree-ment can be approximated as: Half-width ≈ t(n − 1, 0.975) × sd × √[1/n + (1.96^2^/(2(n − 1)))] where sd represents the expected standard deviation of the paired differences. Based on conservative assumptions from pilot observations and previous literature, the standard deviation of the paired differences was assumed to be approximately 0.40°. The target precision was set to a CI half-width of ≤ ±0.20°, corresponding to 10% of the predefined clinically acceptable agreement range (±2°) for HKA angle measurements.

Under these assumptions, a minimum sample size of 54 participants was required. To account for potential exclusions and to further improve precision, 65 participants were recruited, of whom 61 completed the full protocol and were included in the final analysis. [10,11].

### 2.4. Measurement Procedure

All assessments were performed in a standardized clinical environment by a trained evaluator. Participants were instructed to stand barefoot in an upright, relaxed position with knees fully extended, feet placed at shoulder width, and arms resting naturally alongside the body. Subjects were asked to look straight ahead to minimize head and trunk rotation.

To ensure accurate landmark visualization, participants wore fitted shorts or undergarments, allowing exposure of the anterior superior iliac spines, the center of the patella, and the midpoint between the medial and lateral malleoli. The standardized posture was maintained for the duration of image acquisition, which lasted less than 30 s per participant.

#### 2.4.1. Standardization and Image Acquisition Conditions

Image acquisition was performed using a fixed camera-to-subject distance of approximately 2 m, as recommended by the system manufacturer. The camera was positioned at a height aligned with the participant’s knee joint to minimize vertical distortion. Ambient lighting conditions were kept consistent throughout the data collection sessions to reduce shadows and improve image contrast.

Special care was taken to minimize sources of measurement error, including parallax effects, excessive limb rotation, or uneven weight distribution. Participants were instructed to distribute weight evenly across both lower limbs and to avoid voluntary muscle contraction during image capture.

#### 2.4.2. System Calibration Procedure

Prior to each measurement session, the PAViR system underwent an automated calibration process to ensure optimal accuracy of the RGB-D depth camera and landmark detection algorithms. The calibration procedure was performed following the manufacturer’s standardized protocol (Settings > Sensor > RGB-D Calibration). This process in-volves automatic verification of the camera’s intrinsic parameters, including focal length, principal point, and radial distortion coefficients, as well as alignment between the RGB and depth sensors [12].

The RGB-D camera employs an infrared depth sensor with time-of-flight technology capable of capturing depth information at a resolution of 512 × 424 pixels and RGB im-ages at 1920 × 1080 pixels. During calibration, the system automatically detects and adjusts the alignment between the color and depth data streams to ensure accurate spatial correspondence of anatomical landmarks. This process typically requires less than 2 min per session and is verified through a visual feedback interface that suggest proper body outline detection [13].

Following calibration, a standardized test scan was performed to verify that the system’s automated body outline matched the anatomical contours of a reference subject, ensuring that skeletal landmark detection was functioning within acceptable tolerance. Only after successful calibration verification were participant measurements initiated.

For Kinovea analysis (version 0.9.5; Kinovea Team, Bordeaux, France), no additional calibration was required, as the software operates directly on 2D images exported from the PAViR system. Kinovea automatically scales measurements based on pixel-to-distance ratios inherent in the standardized image acquisition protocol [14].

### 2.5. PAViR System Description

The Posture Analyzing and Virtual Reconstruction (PAViR) system (commercially known as Moti Physio) is a portable, modular device designed for postural and biomechanical assessment. The system integrates a high-precision RGB-D depth camera capable of capturing three-dimensional data with a spatial resolution of up to 0.1 mm.

PAViR employs artificial intelligence algorithms trained on a clinical database exceeding 37,000 records, enabling automated detection of anatomical landmarks and computation of biomechanical parameters. Image acquisition is performed in the three anatomical planes, with frontal plane images used in this study for coronal alignment assessment. Standard image resolution was set at 1920 × 1080 pixels.

**Figure 1:**
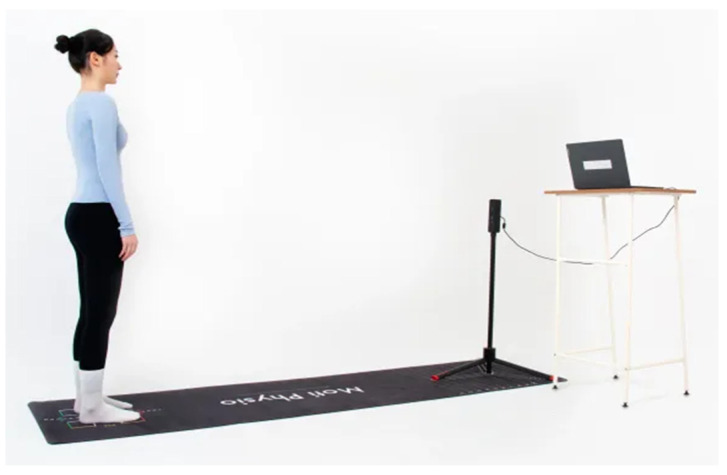
Standard setup of the PAViR system showing subject positioning and camera placement. Image adapted from Ortomecanica [7].

The compact dimensions (approximately 40 × 61 × 20 cm) and weight below 5 kg facilitate deployment in outpatient, rehabilitation, and sports medicine settings. The system connects to standard operating systems via USB or wireless protocols, allowing real-time processing and visualization (Figure 1).

#### Landmark Identification Strategy and AI-Based Detection Algorithm

The PAViR system employs a deep learning-based human pose estimation (HPE) algorithm for automatic detection of anatomical landmarks. The algorithm utilizes RGB-D data captured by the depth camera to identify skeletal key points on the body surface through a multi-stage convolutional neural network (CNN) architecture.

The landmark detection process operates through a bottom-up approach consisting of two sequential learning branches: (i) landmark position prediction, which generates confidence heatmaps for each anatomical key point, and (ii) part affinity field (PAF) computation, which establishes spatial relationships between associated landmarks. This dual-branch architecture enables the system to distinguish between adjacent anatomical structures and maintain geometric consistency across detected landmarks.

The neural network was pre-trained on a proprietary clinical database exceeding 37,000 postural assessment records, encompassing diverse body morphotypes, age groups, and postural configurations. The training process utilized annotated ground-truth data where anatomical landmarks were manually labeled by trained clinicians. The network learns to predict landmark positions by generating Gaussian-distributed confidence heatmaps centered on each key point, with peak values indicating the most probable an-atomical location.

For HKA angle computation, the system identifies three key anatomical landmarks in the frontal plane: the anterior superior iliac spine (hip reference), the center of the patella (knee reference), and the midpoint between the medial and lateral malleoli (ankle reference). These landmarks correspond to those traditionally used in radiographic and photogrammetric alignment assessments.

The detection process is fully automated and requires no manual correction under standardized acquisition conditions. However, the system incorporates a visual feedback interface that allows the operator to verify that the automatically detected body outline and skeletal key points align with the participant’s actual anatomy before finalizing the measurement. This verification step ensures quality control but does not involve manual landmark repositioning. The system’s reported landmark detection accuracy exceeds 86% for correctly positioned key points within a normalized distance threshold, as established during the network validation phase.

The use of AI-assisted landmark detection aims to reduce observer-dependent variability and enhance reproducibility compared to manual photogrammetric methods. Nevertheless, the system’s performance remains influenced by factors such as posture standardization, image quality, lighting conditions, and body surface visibility, which were controlled through standardized protocols as described in Section 2.4.1 [15].

### 2.6. Reference Measurement Using Kinovea

The same frontal images acquired by the PAViR system were exported and analyzed using Kinovea software (version 0.9.5), a free and open-source tool widely employed in clinical biomechanics and sports science research. Kinovea allows precise 2D photogrammetric measurement of angles and distances through manual placement of reference points and angle vectors.

For each image, the evaluator manually identified the anterior superior iliac spine, the center of the patella, and the midpoint between the malleoli. Angle lines connecting these landmarks were drawn to calculate the HKA angle for both lower limbs. All measurements were performed by a single trained evaluator to eliminate inter-observer variability.

**Figure 2:**
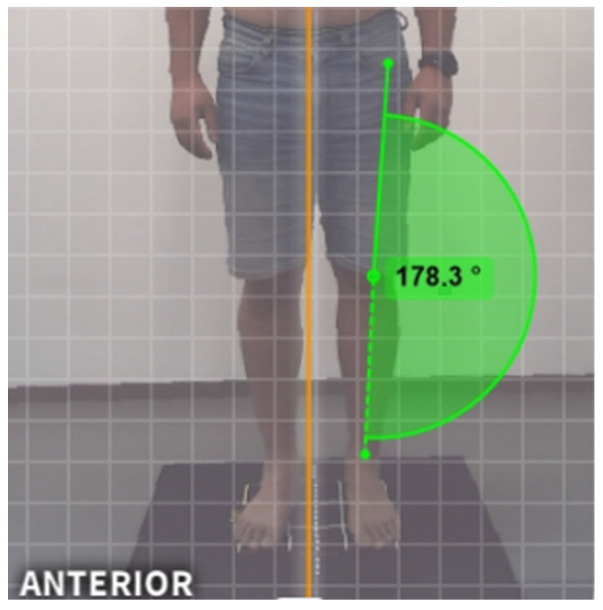
Interface of Kinovea software used for 2D kinematic analysis.

Kinovea has demonstrated strong intra- and inter-rater reliability in previous validation studies, supporting its use as a reference method for 2D angular measurements [6]. Figure 2 explains the interface of the Kinovea software during the HKA angle measurement process. The anatomical landmarks and angle lines are manually placed by the evaluator to ensure accuracy.

To assess intra-observer reliability, a random subset of 15 participants (24.6% of the total sample) was re-measured by the same evaluator one week after the initial assessment, with the evaluator blinded to the previous measurements. Intra-class correlation coefficients (ICC) were calculated using a two-way mixed-effects model for absolute agreement (ICC_3_,_1_). Intra-observer reliability for Kinovea measurements was excellent, with ICC values of 0.996 (95% CI: 0.989–0.999) for the left lower limb and 0.997 (95% CI: 0.991–0.999) for the right lower limb, indicating highly consistent measurements by the evaluator.

### 2.7. Statistical Analysis

Statistical analyses were conducted using IBM SPSS Statistics version 29.0 (IBM Corp., Armonk, NY, USA). Normality was assessed on the paired differences between methods (PAViR − Kinovea), as required for the Bland–Altman approach, using the Shapiro–Wilk test. Descriptive statistics were calculated for all variables, including mean values and standard deviations.

To evaluate the linear association between HKA angle measurements obtained with PAViR and Kinovea, Pearson correlation coefficients were computed along with their 95% confidence intervals. Agreement between the two measurement methods was further assessed using Bland–Altman analysis, allowing identification of systematic bias and estimation of limits of agreement.

A clinically acceptable agreement threshold of ±2° was adopted based on previous literature addressing lower limb alignment variability. Statistical significance was set at *p* < 0.05 for all analyses.

## 3. Results

### 3.1. Descriptive Statistics and Normality

A total of 61 participants completed the full evaluation protocol without adverse events or protocol deviations. Descriptive statistics for the Hip–Knee–Ankle (HKA) angle measured with both the PAViR system and the Kinovea software are presented in Table 1. Mean HKA values obtained with both methods were highly comparable for both lower limbs.

For the left lower limb, the mean HKA angle measured using PAViR was 179.92° (SD ± 3.63), while Kinovea yielded a mean value of 179.89° (SD ± 3.63). For the right lower limb, mean values were 180.53° (SD ± 3.39) for PAViR and 180.49° (SD ± 3.40) for Kinovea. The observed ranges were similar across methods, indicating consistent measurement behavior throughout the full spectrum of alignment values recorded in the sample.

Normality assessment using the Shapiro–Wilk test support that the paired differences between PAViR and Kinovea measurements followed an approximately normal distribution (*p* > 0.05), supporting the use of parametric Bland–Altman limits of agreement.

### 3.2. Correlation Analysis Between Measurement Methods

Pearson correlation analysis revealed an almost perfect linear relationship between HKA angle measurements obtained using the PAViR system and those derived from Kinovea software. For the left lower limb, the correlation coefficient was r = 0.999 (*p* < 0.001; 95% CI: 0.998–1.000). Similarly, for the right lower limb, the correlation coefficient was r = 0.999 (*p* < 0.001; 95% CI: 0.998–1.000), as detailed in Table 2.

These results indicate a remarkably strong association between the two measurement methods across both limbs, with minimal dispersion around the line of identity. The consistency of correlation coefficients for left and right sides suggests that the performance of the PAViR system is not influenced by laterality and that its measurements closely mirror those obtained with the reference method.

While correlation analysis alone does not imply measurement interchangeability, the extremely high correlation values observed provide initial evidence of strong con-current validity between PAViR and Kinovea for static HKA angle assessment. It is critictal to emphasize that high correlation coefficients indicate strong linear association but do not confirm that the two methods can be used interchangeably. Agreement analysis using the Bland-Altman method is the appropriate statistical approach to assess whether two measurement methods produce clinically equivalent results and may be considered interchangeable. For this reason, the Bland-Altman analysis presented in Section 3.3 represents the primary evidence for method agreement.

### 3.3. Agreement Analysis

Agreement between the PAViR system and Kinovea was further evaluated using Bland–Altman analysis. For the left lower limb, the mean difference between methods was 0.03° (SD = 0.14°), while for the right lower limb the mean difference was 0.04° (SD = 0.17°), indicating minimal systematic bias in either direction. The 95% confidence inter-vals for the mean differences were [−0.08°, 0.14°] for the left limb and [−0.05°, 0.13°] for the right limb, demonstrating that the observed differences are not statistically significantly different from zero.

**Figure 3:**
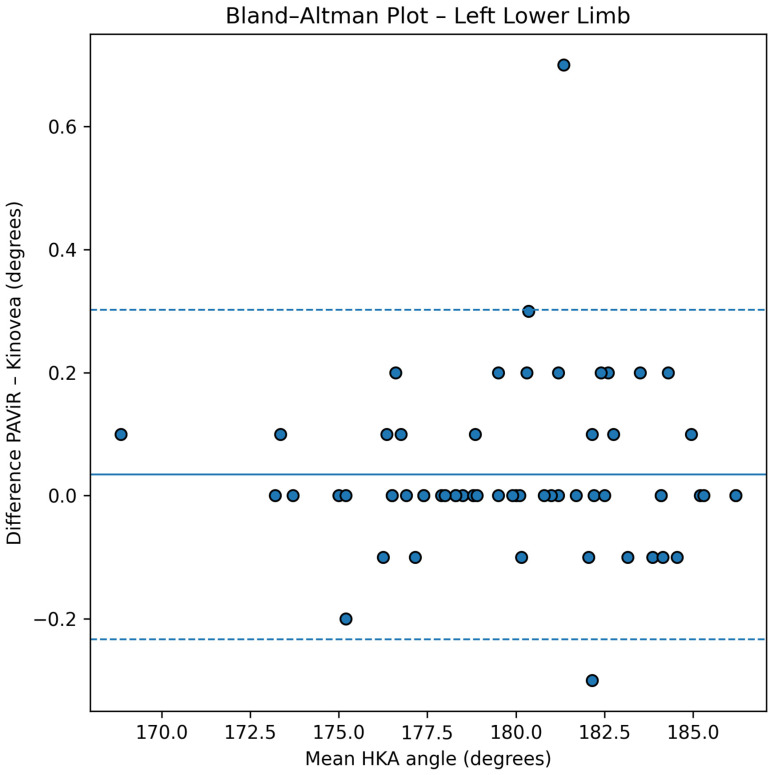
Bland–Altman plot for the left lower limb. The x-axis represents the mean HKA angle obtained from PAViR and Kinovea, and the y-axis represents the difference between methods (PAViR − Kinovea). The solid line indicates the mean bias, dashed lines represent the 95% limits of agreement, and dotted lines denote the 95% confidence intervals for the bias and the limits of agreement.

**Figure 4:**
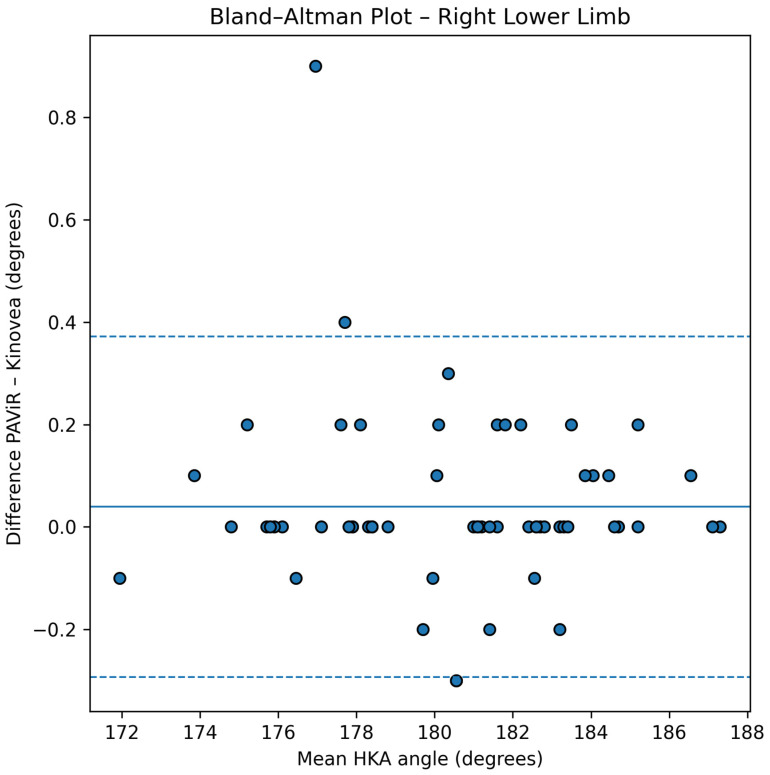
Bland–Altman plot for the right lower limb. Plot configuration and interpretation are identical to Figure 3, showing agreement between PAViR and Kinovea measurements with corresponding mean bias, limits of agreement, and 95% confidence intervals.

The limits of agreement ranged from −0.23° to +0.30° for the left lower limb and from −0.29° to +0.37° for the right lower limb. As shown in Figure 3 and Figure 4, all observed differences fell within these narrow limits for both limbs, indicating 100% concordance with statistical expectations. Notably, all 61 participants’ measurements (122 limbs in total) had differences well below the predefined clinically acceptable threshold of ±2°, with the maximum observed absolute difference below 0.40°, representing less than 20% of the predefined clinically acceptable tolerance.

The Bland–Altman plots reveal a uniform distribution of differences across the entire range of HKA angle values measured (168.8° to 187.3°), with no evidence of heteroscedasticity or proportional bias. The absence of systematic trend across measurement magnitudes indicates that agreement between the two methods is consistent regardless of lower limb alignment configuration (varus, neutral, or valgus). This consistency of agreement across the full measurement range substantially strengthens the validity of the PAViR system for clinical use.

The observed standard deviation of the paired differences (0.14–0.17°) was substantially lower than the conservative value assumed during study planning (0.40°). Consequently, the achieved precision of the estimated limits of agreement exceeded the a priori design target.

### 3.4. Summary of Measurement Performance

Taken together, the descriptive statistics, correlation analysis, and agreement assessment demonstrate that the PAViR system produces HKA angle measurements that are highly consistent with those obtained using a validated 2D reference method. The low mean differences, narrow limits of agreement, and absence of systematic bias indicate that PAViR can reliably reproduce static coronal plane alignment measurements under standardized conditions.

## 4. Discussion

### 4.1. Interpretation of the Validation Findings

The primary aim of this study was to validate the Posture Analyzing and Virtual Reconstruction (PAViR) system as a non-invasive tool for measuring the Hip–Knee–Ankle (HKA) angle in asymptomatic adults. The results demonstrated an almost perfect correlation between PAViR and the reference method, Kinovea [6], along with minimal mean differences and narrow limits of agreement. These findings indicate that, under the controlled experimental conditions of this study, PAViR is capable of reproducing coronal plane alignment measurements with a level of agreement that is well within clinically acceptable thresholds when compared against a validated 2D photogrammetry reference method.

It is important to note that the exceptionally narrow limits of agreement observed in this study (approximately −0.23° to +0.30° for the left limb and −0.29° to +0.37° for the right limb) likely reflect both the technical precision of the PAViR system and the highly controlled experimental conditions under which the validation was performed. The use of asymptomatic participants with normal body habitus, standardized positioning proto-cols, consistent lighting, and a single trained evaluator all contribute to minimizing measurement variability. In real-world clinical settings with diverse patient populations, variable environmental conditions, and multiple operators, the limits of agreement may be wider. Nevertheless, the baseline performance established in this study provides a rigorous foundation for future validation in more heterogeneous clinical contexts.

### 4.2. Comparison with Existing Imaging and Biomechanical Assessment Methods

The findings of this study are consistent with previous research investigating non-radiographic and image-based approaches to lower limb alignment assessment. Prior studies have reported that 2D photogrammetry systems, when applied under standardized conditions, can achieve accuracy levels comparable to radiographic measurements for static alignment parameters [11,12,13]. Similarly, markerless motion capture and depth camera–based systems have demonstrated promising reliability for lower limb alignment evaluation, albeit often requiring more complex setups or computational resources [14,15,16,17,18,19].

Compared with full-length weight-bearing radiography, the PAViR system offers a fundamentally different balance between accuracy, safety, and accessibility. While radiographic assessment remains indispensable for surgical planning in complex cases, its routine use is limited by radiation exposure, cost, and availability. The present results suggest that PAViR demonstrates sufficient agreement for potential use in contexts where repeated non-invasive measurements are required. However, formal validation in clinical populations and longitudinal settings is necessary before recommending its routine implementation in rehabilitation monitoring, sports screening, or preventive musculoskeletal assessment.

Furthermore, when compared with traditional marker-based motion capture systems, PAViR provides a more streamlined and clinically practical solution. Marker-based systems, although highly accurate, are time-consuming, require specialized personnel, and are generally confined to laboratory environments. In contrast, AI-assisted photogrammetry enables rapid data acquisition with minimal setup, making it more suitable for everyday clinical use.

### 4.3. Clinical and Practical Implications

If further validated in diverse clinical populations, non-invasive tools for HKA angle measurement may offer potential clinical utility. Lower limb alignment is a key determinant of joint loading patterns and has been strongly associated with the development and progression of knee osteoarthritis, particularly in varus and valgus malalignment [17,18,19]. However, the present study’s validation was limited to asymptomatic individuals under controlled conditions. Additional research is required to establish whether PAViR can reliably detect clinically relevant alignment abnormalities in pathological populations, and whether such measurements can inform therapeutic decision-making regarding orthotic, rehabilitative, or exercise-based interventions.

The portability and ease of use of the PAViR system suggest potential for deployment in various clinical environments. However, real-world implementation would re-quire additional validation studies to assess performance across diverse settings, user groups, and patient populations, as well as health economic evaluations to determine cost-effectiveness compared to existing assessment methods.

From an imaging perspective, the use of AI-assisted photogrammetry aligns with current trends toward digitalization and automation in musculoskeletal diagnostics. The incorporation of such technologies may contribute to more standardized and reproducible assessments, reducing operator dependency and improving consistency across evaluators and clinical sites [20,21,22].

### 4.4. Methodological Considerations and Limitations

Despite the strengths of the present study, several limitations should be acknowledged.

First, the sample consisted exclusively of asymptomatic adults, which limits the generalizability of the findings to populations with musculoskeletal pathology. Individuals with knee osteoarthritis, severe varus or valgus malalignment, post-surgical conditions, or compensatory postural adaptations may present additional challenges for automated landmark detection and alignment estimation. The presence of soft tissue swelling, joint effusion, skin folds, or altered weight-bearing patterns in pathological conditions could affect the accuracy of photogrammetric landmark identification. Future validation in diverse clinical populations is essential before the PAViR system can be recommended for diagnostic use in orthopedic practice [23].

Second, the assessment was limited to static standing posture. While static alignment provides valuable information for biomechanical analysis, dynamic conditions such as walking, stair negotiation, or single-leg stance may reveal functional alignment abnormalities not captured in static evaluation. Dynamic alignment assessment is particularly relevant for sports medicine applications and functional rehabilitation monitoring. Future studies should explore the validity of the PAViR system under dynamic conditions to further expand its clinical applicability.

Third, all reference measurements were performed by a single evaluator. Although this approach eliminated inter-observer variability, intra-observer reliability was not formally assessed and should be considered in future validation studies. Additionally, while standardized acquisition procedures were implemented, factors such as subtle posture variations or clothing-related landmark visibility may still influence measurement accuracy.

Fourth, the evaluator performing Kinovea measurements was not formally blinded to the PAViR system outputs. Although a standardized measurement protocol was fol-lowed to minimize potential bias, the lack of formal blinding could theoretically influence the reference measurements. Future validation studies should implement double-blinding protocols to eliminate this potential source of bias entirely. Nevertheless, the excellent intra-observer reliability (ICC > 0.99) and narrow limits of agreement suggest that any such bias, if present, was minimal.”

Fifth, the study did not evaluate the PAViR system’s performance under real-world clinical conditions, where factors such as time constraints, diverse patient body habitus, reduced patient cooperation, and variable lighting or spatial conditions may affect measurement accuracy. Controlled laboratory validation, while necessary for establishing baseline performance, does not guarantee equivalent results in routine clinical practice. Sixth, although the study demonstrated strong agreement between PAViR and Kinovea, neither method was directly compared against the gold standard of full-length weight-bearing radiography. Therefore, while the agreement between the two photogrammetry methods is excellent, the absolute accuracy of PAViR for detecting true anatomical alignment remains to be established through comparison with radiographic measurements.

Sixth, although the study demonstrated strong agreement between PAViR and Kinovea, neither method was directly compared against the gold standard of full-length weight-bearing radiography. Therefore, while the agreement between the two photogrammetry methods is excellent, the absolute accuracy of PAViR for detecting true anatomical alignment remains to be established through comparison with radiographic measurements. The present validation suggest technical consistency between two 2D photogrammetry systems but does not substitute for radiographic validation [24,25,26].

### 4.5. Future Research Directions

Future research should aim to validate the PAViR system in clinical populations with known alignment abnormalities, such as patients with knee osteoarthritis, post-surgical conditions, or lower limb deformities [20]. Expanding validation to dynamic tasks, including gait analysis, could further enhance the system’s relevance for functional assessment.

Moreover, integration of real-time feedback and machine learning–based refinement of landmark detection algorithms may further improve accuracy and robustness. Longitudinal studies evaluating sensitivity to change over time would also be valuable to determine the utility of PAViR for monitoring disease progression or treatment outcomes.

## 5. Conclusions

This study demonstrated that the Posture Analyzing and Virtual Reconstruction (PAViR) system shows excellent agreement with a validated 2D photogrammetry reference method (Kinovea) for measuring the Hip-Knee-Ankle (HKA) angle in asymptomatic adults under controlled experimental conditions. The observed minimal mean differences (0.03–0.04°) and narrow limits of agreement (within approximately −0.30° to +0.40° across both limbs) fall well within clinically acceptable thresholds (±2°), supporting the technical validity of PAViR for static coronal plane alignment assessment in this population.

These findings represent a necessary preliminary step in the validation pathway for AI-assisted 2D photogrammetry systems for lower limb alignment assessment. However, several critical validation steps remain before clinical implementation can be recommended. Future research must address: (i) validation in clinical populations with known alignment pathology; (ii) direct comparison with the gold standard of full-length weight-bearing radiography; (iii) assessment of intra- and inter-observer reliability; (iv) evaluation under real-world clinical conditions; and (v) validation in dynamic functional contexts.

Subject to successful completion of these additional validation studies, the PAViR system may represent a feasible non-invasive option for lower limb alignment screening in selected clinical contexts. The present findings provide initial evidence supporting further investigation of this technology for potential integration into musculoskeletal assessment workflows.

## Figures and Tables

**Table 1:** Descriptive statistics of HKA angle measurements with PAViR and Kinovea.

	Descriptive Statistics				
	*N*	Min.	Max.	Mean	SD
Lt_HKAangle_PAViR	61	168.90	186.20	179.9197	3.63189
Rt_HKAangle_PAViR	61	171.90	187.30	180.5295	3.38730
Lt_HKAangle_Kinovea	61	168.80	186.20	179.8852	3.62766
Rt_HKAangle_Kinovea	61	172.00	187.30	180.4902	3.40067
N valid	61				

**Table 2:** Pearson correlation for both lower limbs (PAViR vs. Kinovea).

	Left Lower Limb			Right Lower Limb	
	Lt. HKA angle PAViR	Lt. HKA angle Kinovea	Rt. HKA angle PAViR	Rt. HKA angle Kinovea	
HKA angle PAViR Pearson’s correlation	1	0.999	1	0.999	HKA angle PAViR Pearson’s correlation
Sig. (2-tailed)	–	<0.001	–	<0.001	Sig. (2-tailed)
N	61	61	61	61	N
95% CI	–	[0.998, 1.000]	–	[0.998, 1.000]	95% CI
HKA angle Kinovea Pearson’s correlation	0.999	1	0.999	1	HKA angle Kinovea Pearson’s correlation
Sig. (2-tailed)	<0.001	–	<0.001	–	Sig. (2-tailed)
N				61	61

## Data Availability

The original contributions presented in this study are included in the article. Further inquiries can be directed to the corresponding author. https://doi.org/10.6084/m9.figshare.31332016 (accessed on 2 February 2026).

## Abbreviations

HKA	Hip–Knee–Ankle angle
PAViR	Posture Analyzing and Virtual Reconstruction
2D	Two-dimensional
SPSS	Statistical Package for the Social
